# Temporal fluctuations and geographic distributions of *Leptopilina* (Hymenoptera: Figitidae) species in North Carolina: implications for biological control of *Drosophila suzukii* (Diptera: Drosophilidae)

**DOI:** 10.1093/jee/toaf152

**Published:** 2025-07-10

**Authors:** Kayla M Beckwith, Hannah J Burrack, Martha Flanagan, Gregory Wiggins, Hannah K Levenson

**Affiliations:** Department of Entomology and Plant Pathology, North Carolina State University, Raleigh, NC, USA; Department of Crop and Soil Science, North Carolina State University, Raleigh, NC, USA; Department of Entomology, Michigan State University, East Lansing, MI, USA; North Carolina Department of Agriculture and Consumer Services, Raleigh, NC, USA; North Carolina Department of Agriculture and Consumer Services, Raleigh, NC, USA; Department of Entomology and Plant Pathology, North Carolina State University, Raleigh, NC, USA

**Keywords:** biological control, parasitoid, integrated pest management, spotted-wing drosophila, *Drosophila suzukii*

## Abstract

Biological control is an important component of integrated pest management programs. This can include classical biological control agents, but also adventive biological control agents that naturally established. Here, we provide details of the presence and seasonal biology of potential biological control agents for the invasive *Drosophila suzukii* Matsumura in Southeastern USA blackberry production systems, including a recently established adventive parasitoid wasp species, *Leptopilina japonica* Novković & Kimura. To better understand the populations of this new species, we compare detection methods and report on temporal fluctuations and geographic distributions in North Carolina blackberry fields. In total, we collected 1,142 specimens from 6 sites including 5 different *Leptopilina* species: *Leptopilina boulardi* Barbotin, Carton & Kelner-Pillault, *Leptopilina clavipes* Hartig, *Leptopilina heterotoma* Thomson, *Leptopilina japonica*, and *Leptopilina leipsi* Lue & Buffington. We confirm previous reports of *L. japonica*, *L. boulardi*, and *L. heterotoma* in North Carolina, but report *L. clavipes* and *L. leipsi* in the state for the first time. While several *Leptopilina* species have now been documented in North Carolina, *L. japonica* shows the most promise as a biological control agent for *D. suzukii*. Understanding the when and where each of these parasitoid species is present in crop fields is an important first step in improving *D. suzukii* management efforts. These data will aid in understanding how best to protect these species and how best to incorporate them into on-farm management plans.

## Introduction

Over the next 20 yr, changing environmental conditions, including increasing temperature, increasing CO_2_, and extreme weather events, will threaten our agricultural systems (IPC 2022). These changes will worsen already existing threats—such as nutrient deficiencies, water scarcity, and pest pressure—and instigate new threats—such as redistribution of species populations, including both beneficial and pest species (Grünig et al. 2020, Skendžić et al. 2021, IPC 2022). To address these threats, it will be critical to implement integrated pest management (IPM: Bottrell and Bottrell 1979, Stenberg 2017) and integrated pest and pollinator management (IPPM: Biddinger and Rajotte 2015) principles in agricultural systems.

One key component of IPM and IPPM principles for invasive pest control is the use of classical biological control or the release of predators or parasites from the pest’s native range (DeBach and Schlinger 1964). Concerns surrounding the release of classical biological control agents include non-target effects (Louda et al. 2003) and failed introductions (Goldson et al. 2014). It is estimated that only 34% of classical biological control introductions have been successfully established (Hall and Ehler 1979); of these species, 16% resulted in complete control and 58% in some level of control (Hall et al. 1980). However, when successful, these introductions have the potential to be economically and ecologically beneficial (Pimentel 1965, Hokkanen and Sailer 1985). For example, 2 yr after its introduction, the vedalia beetle (*Rodolia cardinalis*) completely controlled the cottony cushion scale (*Icerya purchasi*), effectively saving California’s citrus industry (Doutt 1958).

In some systems, rather than intentional introductions of biological control agents, adventive biological control agents have established and led to successful control of a pest species. For example, an adventive population of the parasitoid *Trissolcus japonicus* was discovered in 2014 in Maryland, USA prior to any releases (Milnes et al. 2016); this species is an important biological control agent for the brown marmorated stink bug, *Halyomorpha halys*, and has since been reported across North America and in some European countries (Dieckhoff et al. 2021). A similar phenomenon is currently occurring with the potential biological control of the invasive *Drosophila suzukii* Matsumura (spotted-wing drosophila) through classical biological control (Daane et al. 2016, USDA 2021, Stahl et al. 2024) as well as adventive biological control agents (Abram et al. 2020, Beers et al. 2022, Gariepy et al. 2024).

*D. suzukii*, an invasive fruit fly from Asia, was first detected in the United States in California in 2008 and quickly spread across the United States (Hauser 2011). Due to its large, serrated ovipositor, *D. suzukii* is able to attack the ripening stages of all major berry and cherry crops, including blackberries, raspberries, blueberries, strawberries, and cherries (Asplen et al. 2015). Even now, 17 yr after its initial detection, *D. suzukii* causes millions of dollars of damage in lost yield each year (Tait et al. 2021). As concerns about management costs and pesticide resistance increase in *D. suzukii* (Ganjisaffar et al. 2022a, 2022b, Isaacs et al. 2022), alternative control tactics are needed to improve the sustainability and effectiveness of management programs. One promising tactic is the use of biological control agents.

In 2022, while evaluating the release of a classical biological control agent (*Ganaspis kimorum* Buffington) an adventive parasitoid species, *Leptopilina japonica* Novković & Kimura, was detected across North America (Gariepy et al. 2024). While other parasitoid species are present in the United States (Lue et al. 2016, Lee et al. 2019), these species do not effectively parasitize *D. suzukii*. However, previous work has already demonstrated that one species, *L. japonica,* will parasitize *D. suzukii* and shows promise for providing effective aid in pest control (Wang et al. 2020, Nair and Peterson 2023, Neupane et al. 2024). To effectively incorporate this species into management programs, many basic questions need to first be addressed such as their local geographic distributions and temporal fluctuations, which is our focus here.

We first provide a comparison of three sampling and monitoring methods. After identifying the best method for our efforts, we documented temporal fluctuations across one calendar year. Finally, we collected samples from across North Carolina to understand the extent of the geographic distribution of these newly detected parasitoid species. Understanding when and where *D. suzukii* parasitoids are present is critical information that will act as a backbone for updating *D. suzukii* management recommendations.

## Materials and Methods

**Fig. 1.**
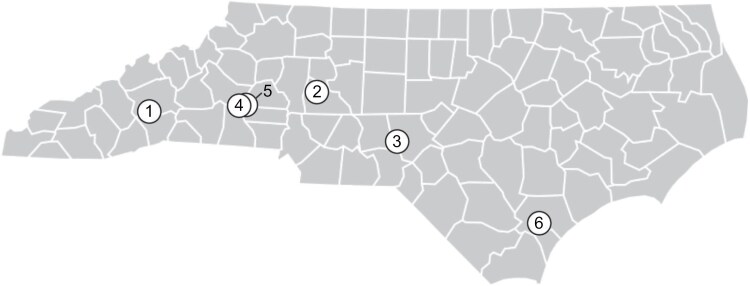
Map of sampling locations using the site numbers detailed in Table 1.

In 2022, the first field releases of the classical biological control agent, *Ganaspis kimorum*, were conducted at Experimental Agricultural Research Stations owned and operated by the North Carolina Department of Agriculture & Consumer Services and North Carolina State University (hereafter referred to as research stations). While conducting these releases and evaluating their success, we unexpectedly found that an adventive parasitoid species, *Leptopilina japonica*, had become established in North Carolina (Gariepy et al. 2024). Following this discovery, we evaluated which of the ongoing monitoring methods was best suited to detecting this species (Table 1 and Fig. 1): liquid traps, fruit incubation, and sentinel traps.

**Table 1.** Summary of the type. location, and region of each location where samples were collected as well as a summary of the sample collection activities conducted at each location.

Site number	Location type	County	Sample collection activities
1	Research Station	Henderson	Comparison of Detection Methods, Geographic Distribution
2	Research Station	Rowan	Comparison of Detection Methods, Additional Detection Method Testing, Temporal Fluctuations
3	Research Station	Moore	Comparison of Detection Methods
4	Commercial Farm	Lincoln	Additional Detection Method Testing, Geographic Distribution
5	Commercial Farm	Cleveland	Geographic Distribution
6	Commercial Farm	Pender	Additional Detection Method Testing

### Comparison of Detection Methods

#### Liquid Traps

We used homemade traps made out of 32 oz deli cups (946.4 ml) with roughly 8 mm wide holes made along the top edge that allowed insects to enter the trap. In the field, traps were hung on a stake (approximately 1.5 m high) with a thin rope at the crop field edge adjacent to the nearest natural area. Traps were filled about one third with a 50:50 mixture of water and propylene glycol antifreeze (Prestone, Des Plaines, IL). A synthetic lure (Scentry Biologicals, Billings, MT) was hung from the lid of each trap with a twist-tie so that it was elevated just above the liquid. Liquid traps, of this or a similar design, are regularly used to monitor for the presence of *D. suzukii* adults in berry and cherry fields (Lee et al. 2013), but we had previously noted that *Leptopilina* spp. were also present, at times in high numbers, in these traps (Gariepy et al. 2024). Each month, one liquid trap per site was hung at the edge of a natural area adjacent to a blackberry field. Once per month, the collected samples were transported back to the Specialty Crops Integrated Pest and Pollinator Management (SCIPPM) Laboratory at North Carolina State University for processing. *Leptopilina* specimens were identified to species, when possible, using the key developed by Lue et al. (2016), and other parasitoid species were identified to the lowest level possible using Abram et al. (2022).

#### Fruit incubation

Once per month, we collected samples of crop fruit, ornamental fruit, and wild fruit from in and around blackberry fields at three research stations. Samples ranged from an average of 61.6 ± 81.4 individual fruit, weighing 18.4 ± 22.7 g (see Levenson et al. 2025), depending on fruit type, size, and availability. We transported samples of each fruit type in separate 8 or 16 oz (236.6 and 473.2 ml, respectively) deli cups, depending on fruit size, back to the SCIPPM lab and held them in an incubator at 25° C for 32 ± 6 d to allow all parasitoid species of interest to emerge using a modified container from Abram et al. (2022). After, collected parasitoid specimens were again identified down to species when possible.

#### Sentinel Traps

Once per month, 12-13 whole blueberries and three banana slices were pre-infested with *D. suzukii* at the Beneficial Insects Lab of the North Carolina Department of Agriculture and Consumer Services’ Biological Control Program. The infested fruit were then held in 8 oz deli cups (236.6 ml) containers with 830 micron mesh inserts in the lids, placed in an orange delta trap (Trécé Incorporated, Adair, OK), and hung on a stake (approximately 0.8 m high) at the edge of a natural area immediately adjacent to a blackberry field to allow parasitism from wild parasitoids. Each month, six sentinel traps were used, three traps containing blueberries and three traps containing bananas, at each of the three research stations (*n* = 36 traps). Traps were left in the field for 4 d; the fruit was then transported to the SCIPPM lab where they were incubated and insects identified following the methods detailed above.

#### Additional Detection Method Testing

Based on our experience using these three sampling methods (see below) we collected additional samples in 2023 (Table 1 and Fig. 1) during peak harvest season from both commercial and non-commercial blackberry fields. To collect these additional samples, we used only liquid traps and fruit incubation. At each site, we set up liquid traps following the same procedure as detailed above, however, the sampling period differed. During the harvest period (May to July), the trap fluid and captured insects were collected weekly. This fluid was transported back to the SCIPPM lab where the collected parasitoids were identified as detailed above. Lures in the traps were replaced every other week. While the lures are labeled to last between 4 and 6 wk, we regularly recommend replacing the lures every other week while monitoring on commercial farms as lure attractiveness can decrease over time. We hung four liquid traps at the crop row edges immediately adjacent to a natural area, with one row separating each trap. We also collected two types of fruit samples every other week: (1) from blackberry crop field edges immediately adjacent to a wooded edge or natural area, and (2) wild fruit from field edges, wooded edges, or natural areas adjacent to the sampled blackberry crop field. These samples were transported back to the SCIPPM lab for processing. When incubating samples in 2023, we added additional modifications to the containers from Abram et al. (2022) following Van Timmeren et al. (2025) and added yellow sticky cards inside the containers to collect emerging insects. Collected parasitoids were again identified as detailed above.

### *Temporal Fluctuations of* Leptopilina *Species*

To understand the temporal fluctuations of different *Leptopilina* species in Southeastern blackberry fields, we compared samples collected via liquid trap (Table 1 and Fig. 1) for one year from October 2022 to November 2023. Liquid trap sampling was conducted weekly, during the harvest period from May 2023 to August 2023, as detailed above. Outside of the harvest period (October 2022 to April 2023 and August 2023 to November 2023), the fluid and captured insects were collected and the lure was replaced monthly. We report all data at the month level. We hung four liquid traps at the row edges as detailed above. Once transported back to the lab, samples were processed and identified as outlined above.

### *Geographic Distribution of* Leptopilina *Species in Southeastern Blackberry Fields*

To understand the geographical distribution of *Leptopilina* species across North Carolina blackberry fields, we collected samples using liquid traps, as detailed immediately above for temporal sampling. From November and December 2022, we collected samples from one site (Table 1 and Fig. 1), and from January 2023 to November 2023, we collected samples from four sites (Table 1 and Fig. 1).

### Data Analysis

Analyses were conducted in R Studio (version 4.5.2 (2025-04-11); R Core Team 2025) using the *lme4* (Bates et al. 2015) and *MASS* (Venables and Ripley 2002) packages. We only used parasitoids that could be identified to species in the analysis and reporting. We used generalized linear mixed-effects models with a negative binomial response and the log link function.

We first investigated if average monthly temperatures and humidity levels (as separate predictor variables) from October 2022 to November 2023 were related to total monthly parasitoid captures (as the dependent variable). We included the site as a random effect. This weather data was pulled from the closest weather station to each site from the State Climate Office of North Carolina, NC State University (2025). We only considered liquid trap captures during this analysis.

We evaluated if the total number of parasitoids, the number of parasitoid species, and the number of *L. japonica* collected (as separate dependent variables) differed by detection method (liquid trap, fruit incubation, and sentinel trap; as the predictor variable), with site as a random effect. We also evaluated if different fruit types (sentinel trap—blueberry, sentinel trap—banana, crop, wild; predictor variable) resulted in different total number of parasitoids, number of parasitoid species, and number of *L. japonica* collected (as separate dependent variables). We used the same models as detailed above and compared outputs using the emmeans package (Lenth 2025). We also evaluated if fruit weight or fruit number (predictor variables) better described our data through AIC comparison using the same models as already outlined. For this analysis, we removed sentinel trap samples as fruit weight was not documented during the preparation of this detection method.

We report temporal fluctuations and geographical distribution of parasitoids descriptively, only, below. Figures were generated using *ggplot2* (Wickham 2016), *ggbreak* (Xu et al. 2021), and *dplyr* (Wickham et al. 2023a) packages.

## Results

**Fig. 2.**
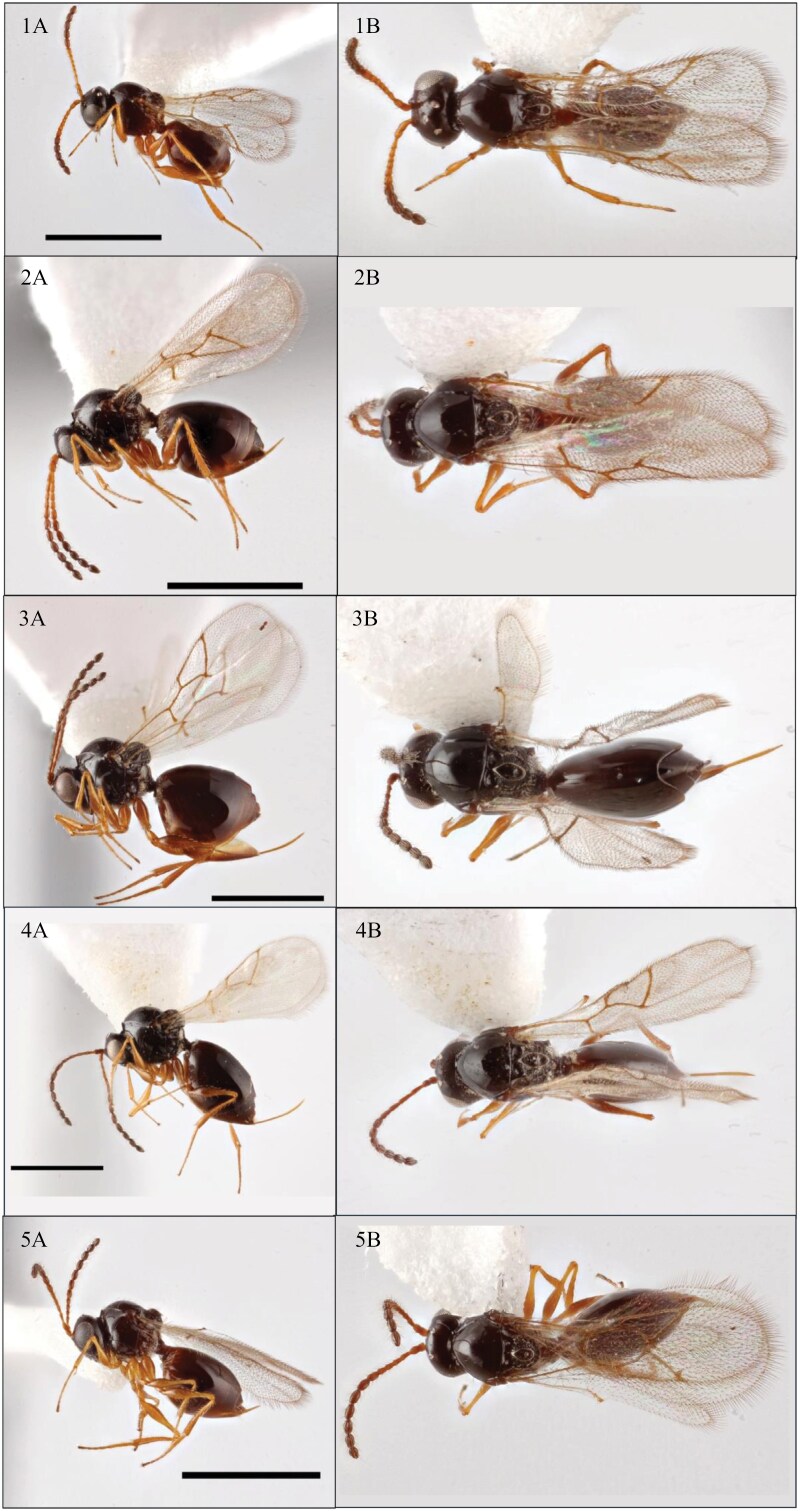
Photographs of representative specimens of the five *Leptopilina* species detected in North Carolina in 2022 and 2023: *Leptopilina boulardi* (1A and B), *Leptopilina clavipes* (2A and B), *Leptopilina heterotoma* (3A and B), *Leptopilina japonica* (4A and B), and *Leptopilina leipsi* (5A and B). Photograph credit: Matt Bertone at NC State University. All ‘A’ photographs show a side angle of the specimen and all ‘B’ photographs show a dorsal angle of the specimen.

A total of 1,142 parasitoid specimens were collected across all sites and all sampling methods (Table 2). We identified 5 different species of *Leptopilina* in North Carolina (Fig. 2) including *Leptopilina boulardi* Barbotin, Carton & Kelner-Pillault, *Leptopilina clavipes* Hartig, *Leptopilina heterotoma* Thomson, *Leptopilina japonica*, and *Leptopilina leipsi* Lue & Buffington. We report *L*. *clavipes* and *L*. *leipsi* for the first time in North Carolina; *L. boulardi*, *L. heterotoma*, and *L. japonica* were previously reported in North Carolina by the authors of this paper (Gariepy et al. 2024). Between October 2022 and November 2023, the average monthly temperature across all sites ranged from a minimum of 3.23 °C and a maximum of 27.03 °C. The average monthly humidity levels across all sites during this time period ranged from a minimum of 57.45% and a maximum of 75.62%. Neither of these weather measurements were significantly related to total monthly parasitoid captures (all *P-values* > 0.69).

**Table 2.** Detailed summary of the abundance of each parasitoid species collected with each sampling method. The locations where each species was detected is noted in the bottom row.

Sampling Focus	Year	Sample Method Type	*Leptopilina boulardi*	*Leptopilina clavipes*	*Leptopilina heterotoma*	*Leptopilina japonica*	*Leptopilina* *leipsi*	*Pachycrepoideus vindemiae*	*Trichopria drosophilae*	*Kleidotoma* spp.
Sampling Method Comparison	2022	Liquid Trap	2	0	1	51	0	1	0	0
		Wild Fruit	0	0	0	7	0	0	0	0
		Sentinel Trap	0	0	2	67	0	0	0	0
	2023	Liquid Trap	3	6	3	92	1	0	6	0
		Wild Fruit	0	0	0	3	0	0	0	0
		Crop Fruit	0	0	3	30	0	0	0	0
Temporal Fluctuations	2022, 2023	Liquid Trap	6	5	16	361	2	0	9	2
Geographic Distributions	2023	Liquid Trap	81	7	23	340	8	0	2	0
Sampling Location Where Detected			Sites 1, 2, 4, 5, 6	Sites 1, 2, 4, 5	Sites 1, 2, 3, 4, 5	Sites 1, 2, 3, 4, 5, 6	Sites 1, 2, 4, 5	Site 1	Sites 2, 4, 5	Site 2

### Comparison of Detection Methods

All detection methods successfully collected some number of parasitoid specimens, with a total of 279 specimens collected across all methods and both years (Table 2). These specimens included *L. boulardi, L. clavipes*, *L. heterotoma, L. japonica*, *L. leipsi*, *Trichopria drosophilae* Perkins (Hymenoptera: Diapriidae), and *Pachycrepoideus vindemiae* Rondani (Hymenoptera: Pteromalidae).

In 2022, a total of 55 specimens were collected using liquid traps, 7 from fruit incubation, and 69 from sentinel traps. Parasitoid wasps were collected in 3 out of 6 liquid traps. Fruit types that were collected for incubation included blackberry, raspberry, grape, pokeweed, Chinese privet, bittersweet vine, and firethorn (similar to Gariepy et al. 2024; see Levenson et al. 2025). Parasitoid wasps only emerged from 2 of the 12 incubation fruit samples: blackberry and pokeweed. In 2023, during the peak harvest period, a total of 111 specimens were collected using liquid traps, 3 from wild fruit incubation, and 33 from crop fruit incubation. Parasitoids were collected in 44 out of 124 liquid traps. Wild fruit types that were collected for incubation included blackberries, wild strawberry, and pokeweed. Out of 10 wild fruit incubation samples, parasitoids emerged from 3 samples: wild blackberries and wild strawberry. Out of 18 crop fruit incubation samples, parasitoids emerged from 9.

We found that the total number of parasitoids was significantly different across detection methods, with liquid traps collecting more parasitoids (*Z*_201_ = 2.26, *P* = 0.024) when fruit incubation was treated as the reference level. Sentinel traps collected numerically more parasitoids, but this was not significant (*Z*_201_ = 1.34, *P* = 0.18). This pattern is mainly driven by the abundance of *L. japonica* as there were also significantly more *L. japonica* collected by liquid traps (*Z*_201_ = 2.08, *P* = 0.038) but not a significant difference in the number of parasitoid species across detection methods (all *P-values* above 0.18). The total number of parasitoids was also significantly different across fruit types with sentinel trap—bananas collecting more parasitoids than sentinel trap—blueberries (*Z*_64_ = −2.84, *P* = 0.005) and wild fruit (*Z*_64_ = −2.70, *P* = 0.007), but no other comparisons were significant (all *p-values* above 0.25). Sentinel trap—bananas also collected more parasitoid species than blueberries (*Z*_64_ = −1.39, *P* = 0.03), but no other comparisons were significant (all *P-values* above 0.16). Again, this pattern was mainly driven by the abundance of *L. japonica* (sentinel trap—bananas had more *L. japonica* compared to sentinel trap—blueberries (*Z*_64_ = −2.65, *P* = 0.008) and wild fruit (*Z*_64_ = −2.50, *P* = 0.012)). Through AIC comparison, we found that fruit weight better explained the total number of parasitoids collected (AIC = 98.1 for fruit weight and AIC = 102.4 for fruit number), however this measure is less realistic for real-world implementation as it requires extra equipment and fruit number is a more relevant metric for stakeholder groups. There was not a notable difference between fruit weight or fruit number with the number of parasitoid species (AIC = 64.5 for fruit weight and AIC = 66.5 for fruit number) or for the abundance of *L. japonica* (AIC = 95.1 for fruit weight and AIC = 99.6 for fruit number).

### *Temporal Fluctuations of* Leptopilina *Species*

**Fig. 3.**
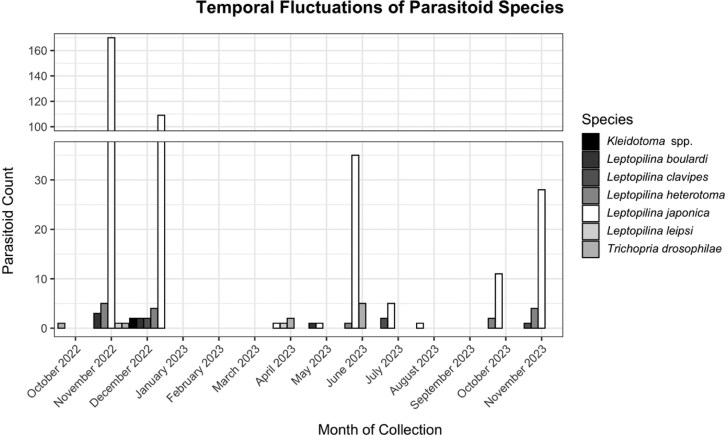
Temporal fluctuations of parasitoid wasp specimens collected by liquid trap at Site 2 from October 2022 to November 2023. The total counts of five *Leptopilina* species are represented as well as *Trichopria drosophilae* and *Kleidotoma* spp.

From October 2022 to November 2023 at Site 2, we collected 401 parasitoid specimens using liquid traps (Table 2 and Fig. 3) including *L. boulardi, L*. *clavipes, L. heterotoma, L. japonica, L*. *leipsi*, *T. drosophilea*, and *Kleidotoma* spp. Westwood (Hymenoptera: Figitidae) (Table 2 and Fig. 3). We report *Kleidotoma* for the first time in NC. We detected parasitoids in 8 mo of the year but detected no parasitoids in January 2023, February 2023, March 2023, and September 2023. We detected the highest abundance of parasitoids in November 2022 (*n* = 181), followed by December 2022 (*n* = 120), and June 2023 (*n* = 41).

### *Geographic Distribution of* Leptopilina *Species in Southeastern Blackberry Fields*

**Fig. 4.**
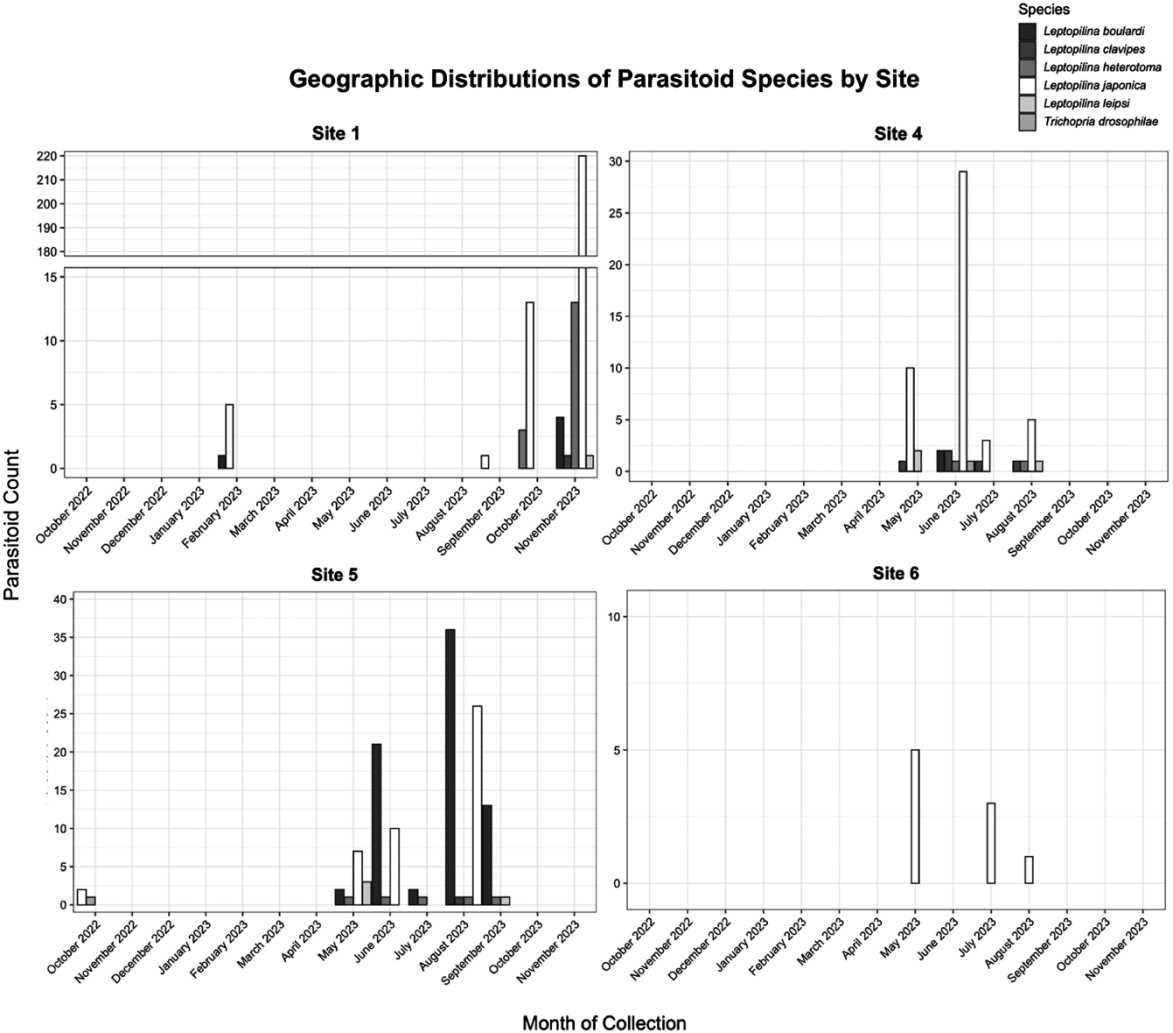
Geographic distribution of parasitoid wasp specimens collected by liquid trap at Sites 1, 4, 5, and 6 from October 2022 to November 2023. Total counts of parasitoids collected per site, with four liquid traps used at all four locations, are shown on the graphs. Five *Leptopilina* species are represented as well as *Trichopria drosophilae*.

Across Sites 1, 4, 5, and 6 (Table 2 and Fig. 4), we collected 461 parasitoid specimens. We collected the most specimens at Site 1 with 277, followed by Site 4 (*n* = 136), then Site 5 (*n* = 60), and the least specimens detected at Site 6 (*n* = 10; Figs. 1 and 4). All four sites detected *L. japonica*, the most out of all *Leptopilina* species. Of the other species, we collected *L. boulardi*, *L. heterotoma*, *L. clavipes*, *L. leipsi*; these species were detected at all sites except for Site 6. We also detected two specimens of *T. drosophila* across all sites (Fig. 4).

Across the four sites, we detected parasitoid wasps during 9 months, November 2022 through November 2023, with no parasitoids detected in November 2022—January 2023, March 2023—April 2023. We found the most parasitoid wasps in November 2023 (*n* = 239). We found six parasitoid wasps in February 2023, 31 in May 2023, 68 in June 2023, 13 in July 2023, 74 in August 2023, 18 in September 2023, and 31 in October 2023.

## Discussion

We show that the adventive parasitoid wasp, *Leptopilina japonica*, is highly abundant and widespread in Southeastern US blackberry fields. At all sites sampled in our study, except for one, *L. japonica* was the most abundant parasitoid wasp species collected. Of note, we report two *Leptopilina* species in the state for the first time (*L. clavipes* and *L. leipsi*) and confirm the presence of four other species previously reported in North Carolina (Lue et al. 2016, Gariepy et al. 2024).

When considering the three monitoring methods, while sentinel traps provide the most detailed information on parasitism and parasitism rates, they require greater inputs of supplies, labor to prepare the fruit before placing them in the field, and labor to process the fruit after collecting from the field. Additionally, this sampling method requires having a laboratory colony of *D. suzukii* and so will not be accessible to everyone. We also note that while using bananas in sentinel traps resulted in more collected parasitoids than blueberries, this fruit type, again, requires greater inputs of labor as this fruit is not specific to *D. suzukii* and instead can collect a wide variety of other fruit fly species and other insects. Collecting wild fruit eliminated the preparation step required in the sentinel traps, making it less labor intensive and more accessible. While this sampling method does provide information on if parasitism is occurring, it does not provide information on the rate of parasitism since the number of flies is not known. Of further consideration, we had very few wild fruit samples that contained emerging parasitoids. While liquid traps only provide information about species presence, not parasitism rates, they required minimal preparation and no post-collection incubation period; they also collected high numbers of parasitoids. Thus, this sampling method was best suited to detecting parasitoids for our purposes.

Across all sampling methods used from 2022 to 2023, parasitoid wasps were active for most of the year except for January 2023, March 2023, and April 2023. The majority of collected parasitoids were collected in May 2023, June 2023, July 2023, August 2023, and November 2023. The temporal abundance of *Leptopilina* species differed across months with *L. japonica* most abundant in February 2023, May 2023, October 2023, and November 2023; and *L. boulardi* most abundant in June 2023, July 2023, and September 2023. These two species were equal in abundance in August 2023. It is of note that we captured high parasitoid abundances late in the year (ie November). One explanation for this occurring, specifically in the Southeastern US, is that as fruit availability decreases going into the winter months, our traps may become more attractive to these parasitoids. Future research should work to better understand the drivers of trap captures at different times of year.

We also detected parasitoid species outside the genus *Leptopilina* including *Trichopria drosophila* (*n* = 9), *Pachycrepoideus vindemiae* (*n* = 1), and 2 specimens in the genus *Kleidotoma.* Both *T. drosophila* and *P. vindemiae* are pupal parasitoids of *D. suzukii* (Hogg and Daane 2024). While previous work has shown that these species will not provide the same level of *D. suzukii* control as *L. japonica*, they may still aid in control to some level (Wang et al. 2020, Hogg et al. 2022, Neupane et al. 2024) and their presence in Southeastern blackberry fields is of note.

As biological control agents in other pest systems have shown significant regional differences across the United States in terms of distribution and effectiveness (Zhu et al. 2024), understanding the temporal fluctuations and geographic distributions of *D. suzukii* parasitoids, early in their establishment, will be a critical component for improving IPM and IPPM practices. These parasitoid wasp species provide differing levels of control (Wang et al. 2020) so using distribution data, such as the data presented here, will aid in understanding what roles different *Leptopilina* species can play in pest management plans. Although the native parasitoid species may not provide sufficient control of *D. suzukii* (Stacconi et al. 2015, Knoll et al. 2017, Sánchez-González et al. 2020), previous research has documented competition between parasitoid species (Hougardy et al. 2022), a non-target effect documented in other biological control systems as well (Louda et al. 2003). Tracking the distribution of parasitoid species, beyond *L. japonica*, will be important for understanding the impact of the recent establishment of *L. japonica* on the populations of other North American parasitoid species. Another consideration is how parasitoids should be incorporated into updated pest management plans. Recent work has shown that some parasitoids are sensitive to pesticides commonly used for *D. suzukii* control (Schlesener et al. 2019, Gao et al. 2024). Care will need to be taken to protect parasitoid wasp populations on farms so that they can provide the critical ecosystem service of additional pest control during active pest management programs. Since these parasitoids have been documented in a variety of cultivated and wild plant types (Gariepy et al. 2024), creating management plans for preserving or augmenting these plants in agricultural areas should be considered as these resources could provide important resources and refugia.

## Data Availability

Data are available via Dryad repository (Levenson et al. 2025; https://doi.org/10.5061/dryad.bcc2fqzr7).
